# labapi: a Python object model for the LabArchives electronic lab notebook

**DOI:** 10.64898/2026.09.02.748406

**Published:** 2026-09-07

**Authors:** Christoph Li, Josh Lawrimore, Dustin Moraczewski, Adam G. Thomas

**Affiliations:** 1 Data Science and Sharing Team, National Institute of Mental Health, National Institutes of Health, Bethesda, MD, USA; 2 Clinical Monitoring Research Program Directorate, Frederick National Laboratory for Cancer Research, Frederick, MD, USA

## Abstract

labapi is a Python library that enables computational workflows to connect to LabArchives’ electronic lab notebook (ELN). Without an Application Programming Interface (API) connection, researchers must manually add workflow outputs through the LabArchives web interface, navigating to the appropriate page and uploading each output so that it appears with the experimental notes that provide context. labapi translates the flat LabArchives API into a Python object model following the existing hierarchy of the web interface, allowing workflows to navigate and modify notebook content through familiar paths.

labapi enables researchers to build interconnected workflows that both read in and write data to LabArchives’ ELN automatically. Researchers can inspect those outputs in the notebook, and later analysis code can read them back for another stage of analysis.

## Statement of need

NIH intramural policy requires new research to be documented in an approved electronic notebook, and LabArchives is one approved option (NIH Office of Intramural Research, 2024). LabArchives is also used by academic and other research organizations outside NIH (LabArchives, LLC, 2026b). Once uploaded to LabArchives, an output becomes part of the laboratory record beside the notes and revision history that document the experiment (LabArchives, LLC, 2026b).

When a pipeline produces an output to be recorded after every run, using the web interface requires the researcher to return to the appropriate page each time. Researchers can automate that transfer through the LabArchives web API, but doing so requires custom integration code. Researchers navigate the web interface by notebook path; API calls require an internal identifier for each page. Code using the API directly must resolve the path to that identifier and construct the requests that read or write the page (LabArchives, LLC, 2026a). labapi performs this work so that researchers can configure workflows with notebook paths. The authors develop two applications built on labapi that other NIMH intramural laboratories use in their research: muronto records neuro-behavioral experiment outputs, while save-my-jupyter deposits Jupyter notebook snapshots (Lawrimore, 2026; Li, 2026). The intended users are computational researchers and research software developers working in or with experimental laboratories that use LabArchives.

## Software design

labapi was designed to simplify working with the LabArchives API and make it familiar to users of the web interface. To provide this familiarity, labapi represents the notebook hierarchy shown in the web interface as Python objects. Moving through this hierarchy is essential to working with a notebook, but difficult through the LabArchives API. Helpers such as dir() and page() ensure that directories and pages exist at a path, retrieving matching nodes or creating missing nodes and parent directories, while traverse() resolves only existing paths (Figure 1).

Additionally, the design follows the maxim “Parse, don’t validate” (King, 2019). labapi parses escaped notebook-path strings into canonical NotebookPath objects that preserve segment and absolute/relative semantics for composition, resolution, containment, and tree traversal.

create_json_entry() stores JSON as an attachment for reuse by code and adds a formatted preview for review in the web interface. A library-level meta-entry was considered, but it would break the direct correspondence between labapi objects and native LabArchives entries. Preserving the grouping for other tools would then require additional bookkeeping entries, so richer metadata schemes were left to higher-level applications.

Applications built on labapi may also need to serve more than one researcher. API credentials are therefore held in a Client, separate from each User session, so a centralized application can retain multiple sessions without distributing the credentials.

## State of the field

Benchling, RSpace, and eLabFTW pair their APIs with official client libraries (Benchling, 2026; eLabFTW, 2026; Research Space, 2026). LabArchives publishes its API without a public official client library. The standalone LabArchives alternatives in the table do not combine path navigation, entry handling, and attachment transfer in a single client (LabArchives, LLC, 2026a):

Among the standalone projects in the table, labarchives-py comes closest. Its single-module implementation signs arbitrary GET requests and returns raw responses, leaving researchers to choose endpoints, parse responses, and implement notebook navigation, entry handling, and file-transfer helpers. Extending it would still have required building the notebook model and higher-level record operations, so labapi was developed as a separate client. Specialized ReDBox and MCP integrations also exist, but neither provides a general-purpose, path-oriented Python notebook object interface (Brudner, 2026; University of Technology Sydney eResearch, 2024).

## Example workflow

In the example, a researcher adds labapi to a two-stage quality-control workflow using five subjects from the public OpenNeuro dataset ds000228 (Richardson et al., 2019). The schematic listings use subjects as a placeholder for the five subject identifiers and compute_qc() and summarize() as placeholders for scientific calculations, so only the high-level LabArchives interaction is shown. Complete setup and entry-handling examples are available in the documentation for working with paths, JSON entries, and example applications. During the first stage, the workflow writes a group label and mean derivative of root mean square variance over voxels (DVARS) for each subject to LabArchives. Mean DVARS is a quality-control value that summarizes how much a functional magnetic resonance imaging (fMRI) scan changes between successive time points (Power et al., 2012). The second stage reads the stored values from LabArchives and produces a cohort summary.

The first stage uses an existing notebook named Lab QC, creates or reuses subject pages under Partly Cloudy QC, and writes each subject’s data as a JSON attachment with a formatted preview.


user = labapi.Client().default_authenticate()
qc = user.notebooks[“Lab QC”].dir(“Partly Cloudy QC”)
for subject in subjects: 
    qc.page(f“sub-{subject}”).entries.create_json_entry(compute_qc(subject))


During the second stage, the workflow opens the existing Partly Cloudy QC folder with traverse(). Each subject page contains the JSON attachment followed by its formatted preview, so the workflow loads the first entry from every page into records.

After loading the five JSON files, summarize() produces a cohort summary and figure. The workflow writes both to the Dashboards/Cohort QC page.


notebook = user.notebooks[“Lab QC”]
qc = notebook.traverse(“Partly Cloudy QC”)
records = [json.load(page.entries[0].content) for page in qc.children]

summary, figure_path = summarize(records)
dashboard = notebook.page(“Dashboards/Cohort QC”)
dashboard.entries.create_json_entry(summary)


Finally, the workflow attaches the figure to the same page.


attachment = labapi.Attachment.from_file(figure_path)
dashboard.entries.create(labapi.AttachmentEntry, attachment)


The resulting LabArchives page is shown in Figure 2.

## Research impact statement

labapi underpins laboratory record-keeping tools in use beyond the authors’ team, including the muronto and save-my-jupyter applications described above (Lawrimore, 2026; Li, 2026). The library’s notebook-backup support was developed in coordination with the Systems Neuroscience Imaging Resource (SNIR), a core facility providing imaging and image-analysis support to NIMH intramural investigators, which uses labapi to automate backups of its LabArchives notebooks (nimh-dsst/labapi contributors, 2026). NIH policy also requires intramural researchers to use an approved electronic notebook for new research (NIH Office of Intramural Research, 2024), and LabArchives publishes no official client library; labapi fills that gap for laboratories that need to automate this record-keeping.

## Figures and Tables

**Figure 1:**
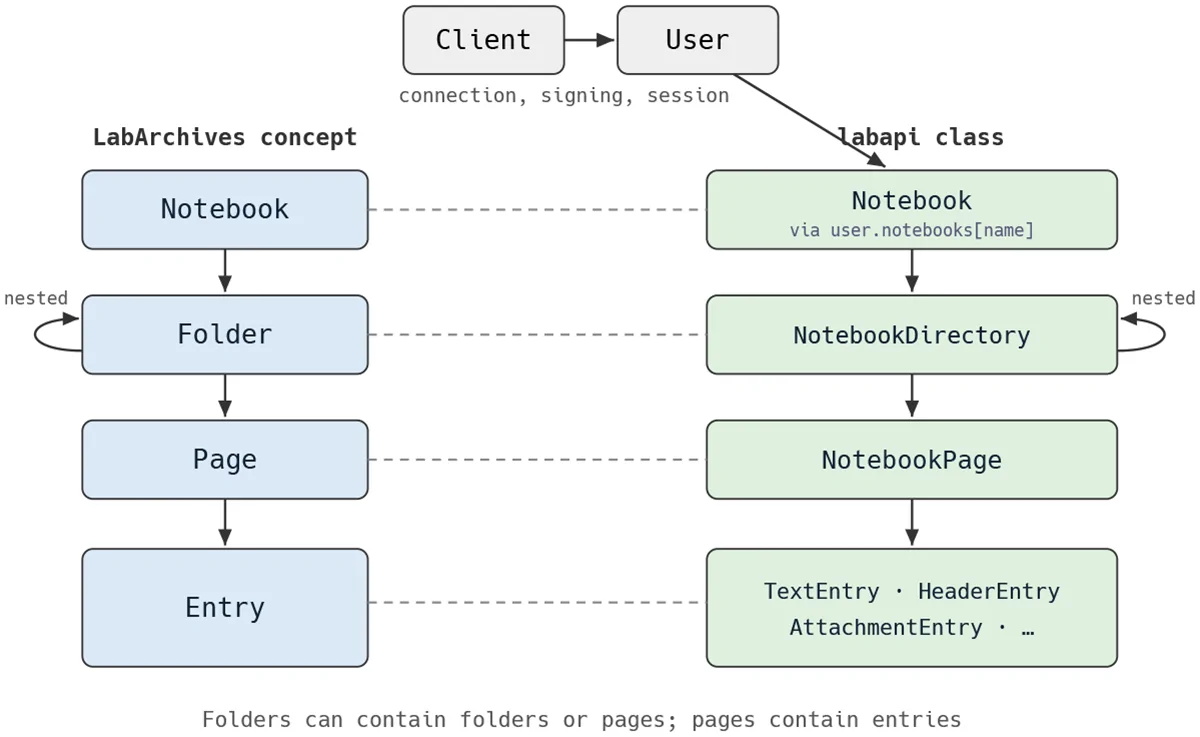
The LabArchives notebook hierarchy and its corresponding labapi objects.

**Figure 2:**
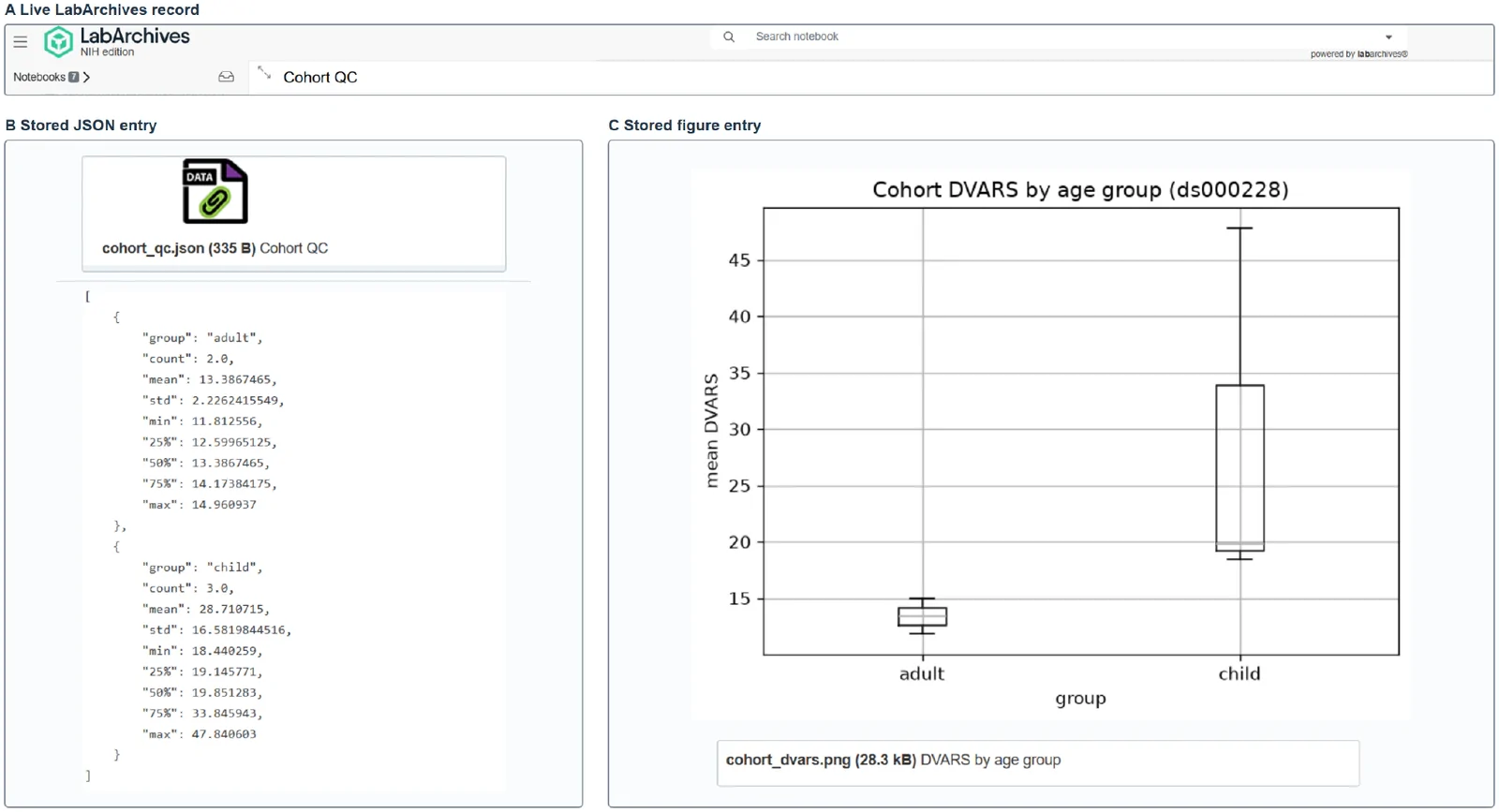
The live LabArchives page containing the cohort summary, a formatted preview of the summary, and the uploaded figure.

**Table 1:** Standalone public LabArchives clients, checked 20 August 2026.

Project	What it provides	Navigable notebook objects
labapi	General Python client	Yes
labarchives-py (Cmero, 2022)	Signed generic GET-call wrapper	No
labarchives-js (Fuschi, 2019)	Login, image-entry search, and attachment-URL helpers	No
labarchives-client (alaninmcr, 2015)	Unmaintained: no released functionality	No

## Data Availability

The source and documentation are available at https://github.com/nimh-dsst/labapi and https://nimhdsst.github.io/labapi/v1.2.0/, respectively. Version 1.2.0 is archived on Zenodo (Li et al., 2026) and released under the MIT License. labapi is also published on PyPI, can be installed with pip install labapi, and requires Python 3.10 or later. The repository includes end-to-end examples, credential-free unit tests, and opt-in live integration tests.
